# Nurses in expanded roles to strengthen community-based health promotion and chronic care: policy implications from an international perspective; A commentary

**DOI:** 10.1186/s13584-018-0257-5

**Published:** 2018-10-12

**Authors:** Claudia B. Maier, Hannah Budde, James Buchan

**Affiliations:** 1 Department of Healthcare Management, Technische Universität Berlin, Straße des 17. Juni 135, Berlin, Germany; 2 Center for Health Outcomes and Policy Research, School of Nursing, University of Pennsylvania, Senior Fellow, Claire Fagin Hall, 418 Curie Blvd, Philadelphia, PA 19104-4217 USA; 3 WHO Collaborating Centre for Nursing, Midwifery and Health Development, Faculty of Health, University of Technology, PO BOX 123, Broadway NSW, Sydney, 2007 Australia

**Keywords:** Health workforce, Community, Nurses, Advanced practice nurses, Health promotion, Chronic disease, Prevention, Implementation, Policy

## Abstract

Chronic conditions and health inequalities are increasing worldwide. Against this backdrop, several countries, including Israel, have expanded the roles of nurses as one measure to strengthen the primary care workforce. In Israel, community nurses work in expanded roles with increased responsibilities for patients with chronic conditions. They also work increasingly in the field of health promotion and disease prevention. Common barriers to role change in Israel are mirrored by other countries. Barriers include legal and financial restrictions, resistance by professional associations, inflexible labor markets and lack of resources. Policies should be revisited and aligned across education, financing and labor markets, to enable nurses to practice in the expanded roles. Financial incentives can accelerate the uptake of new, expanded roles so that all patients including vulnerable population groups, benefit from equitable and patient-centered service delivery in the communities.

## Background

Policy makers in many countries aim to strengthen primary care and community-based services to ensure equitable, accessible and high quality care. Israel and other high-income countries are in the process of introducing changes to their health workforce, often for nurses working in primary care settings and communities. The article by Nissanholtz-Gannot et al. [1] on community nurses in Israel is published at a time where many reforms expanding nurses’ roles are ongoing worldwide [2–4]. In this commentary, we reflect on the situation in Israel in light of international evidence.

### Nurses’ roles in chronic care are expanding in many countries worldwide, in response to increasing patient needs.

In a survey among community nurses in Israel, 85% reported that their nature of work had changed substantially over the recent past (ibid.) [1]. Most changes occurred related to chronic conditions. In Israel, 38% of the community nurses reported that caring for patients with chronic conditions was their main role performed, in addition to routine nursing care. Examples of new tasks commonly reported were the management of the care process, the development of a proactive action plan for patients with chronic conditions and outreach to target populations (80%, 77% and 52% respectively).

Similar developments are also observed in other countries worldwide. In an international study covering 39 countries, two thirds had expanded the scopes-of-practice of nurses in primary care [3]. Chronic conditions and the need to provide a more comprehensive set of services, have become important drivers which led to a new skill-set and task reallocation among nurses within the primary care workforce [2, 5, 6]. For instance, in Australia, Canada, Finland, Ireland, the Netherlands, United Kingdom and the United States (U.S.), Nurse Practitioners (NPs) or other Advanced Practice Nurses (APN) with usually a Master’s degree take care of patients with chronic conditions and have considerably expanded scopes-of-practice [3]. These include the authority to order tests, diagnose/perform advanced health assessments, prescribe (certain) medications and make treatment and referral decisions (ibid.).

Although detailed tasks and roles vary across countries, NPs in these countries can have their own panel of patients or be in charge of specific patient groups, which is important in terms of continuity of care and efficient division of work. In the U.S., NPs in primary care settings work fully independently in some U.S. states as per scope-of-practice laws, which are among the most progressive worldwide. In the remainder U.S. states, a collaborative agreement with a physician is required by law [7, 8]. In Finland, so-called nurse prescribers work in health centers in close collaboration with physicians, and perform routine visits for patients with chronic conditions. Since 2011, these nurses can also prescribe medications on a continued basis for patients with hypertension, type 2 diabetes and asthma, provided they are educated as nurse prescribers and meet other requirements [9].

Overall, many countries in Europe are still early on in the process of expanding the roles of nurses by reforming the educational system, yet, their official scopes-of-practice are sometimes only marginally or not at all expanded. These uneven developments between expanded skills and restrictions to scopes-of-practice, may lead to an inefficient use of nurses’ skills and competencies [2]. Moreover, most countries in Europe do not allow nurses to have their own panel of patients (ibid).

In Israel, two thirds of the health plan nurses working in the community hold academic degrees and many of them have undergone additional training. Most nurses in the survey by Nissanholtz-Gannot et al. [1] had a Bachelor’s degree (47%), and 17% had graduated from a Master’s programme. Additional information about the type of education, curricula and the skills taught is not provided. Although NPs have also been introduced in Israel, with some working in diabetes care, it is not comparable with the situation in the U.S. where NPs work in considerably expanded clinical roles. Further research should investigate the detailed tasks and division of work between physicians, NPs and the community nurses in the delivery of chronic care and identify which skill-mix models are effective and efficient in Israel and other countries worldwide.

### Nurse role expansion in health promotion is evolving in Israel and other countries, but the evidence base is limited, particularly for vulnerable patient groups.

The second most frequently reported set of activities by community nurses in Israel working in new, expanded roles was related to health promotion (30%). Health plan nurses are involved in identifying target populations for health promotion and prevention (86%). Moreover, nurses reported performing additional tasks such as counselling about nutrition, smoking and physical activity (79%, 65% and 73% respectively) [1].

Health inequalities are widening globally. The most vulnerable population groups are having difficulties accessing the health system and receiving patient-centered care. Canada and the U.S. provide relevant experiences on NPs working in advanced roles in communities performing health promotion, health literacy activities or other prevention services, often for vulnerable groups. For instance, NPs have been hired in rural practices in British Columbia, Canada, working with marginalized groups such as patients with HIV/AIDS, with mental health conditions or elderly, frail patients. This has shown improvements in terms of patient access, professional satisfaction and hospital admission rates [10]. In the U.S., NPs and other APN are more likely than physicians to work in rural areas or care for vulnerable patient groups, e.g. uninsured or other marginalized populations and thereby contribute to reducing inequitable access [11–13].

In Israel, public health measures are often performed inside preventive care clinics, such as for early childhood and pregnant women; and to a lower extent in the communities [14]. In the survey by Nissanholtz-Gannot et al. [1], only 14% of the nurses reported performing health promotion activities outside clinics. It is not mentioned which population groups are mainly targeted. Since one third of all nurses in Israel work in the community setting and most of them are employed by one of the non-profit health plans, one strategy to improve access to health promotion and prevention could be to provide additional training and resources to the community nurses in the health plans to perform outreach activities for at risk population groups. Further research should analyse the role of nurses in the provision of prevention services and health promotion activities, and their outcomes in terms of quality of care, costs and equitable access. To date, cross-country comparative research on the expanded role of nurses in health promotion and prevention for vulnerable and at risk population groups is scarce. Particularly, there is limited evidence on how teams including nurses, should be trained effectively and incentivised to provide tailored services to the varied population groups that vulnerable populations often comprise, including the diverse ethnic and socio-economic backgrounds, resources, health literacy and language skills.

Community nurses in Israel have reported a considerable change over time and have taken on more roles and responsibility in chronic care and preventive care, as reported by Nissanholtz-Gannot et al. [1]. Is there a need for further change? In the mid- and longer term, a systematic evaluation of unmet healthcare needs of patients with chronic conditions and underserved populations in particular, may provide evidence if further skill-mix changes are required in the communities. Yet, in the short term, evaluating and removing the barriers to practice that the nurses are already experiencing should be the priority.

## Barriers and enablers to implementation

In Israel, several barriers to community nurses’ abilities to work in expanded roles were identified [1]. These include unclear professional boundaries, opposition by professional associations, as well as low compensation levels, among others. Similar barriers have been reported in other high-income countries. In a systematic review, strict and inflexible professional boundaries, insufficient knowledge and capabilities, unsupportive organizational and institutional environments, were identified as barriers to the uptake of new nursing roles in practice [15]. At the health system level, policies restricting regulations of scope of practice, payment and reimbursement policies were shown as limiting nurse’s potential to work in advanced roles.

### Policies on scopes-of-practice

Restrictive regulation and legislation has been shown to negatively impact on the uptake of expanded nursing roles. In the Netherlands and Spain, initial, restrictive laws were prohibiting nurses to perform certain tasks, e.g. the prescribing of medicines despite the competencies available [16, 17]. New laws were adopted between 2010 and 2015 in these countries allowing nurses to work in expanded roles [2]. In Finland, a 2010 law which was implemented in 2011, authorized nurses to prescribe from a pre-defined list of medicines and allowed nurses to write sickness certificates, among others [9]. In the U.S., restrictive laws were negatively impacting NPs work and access, whereas changes to state regulations expanding the scopes-of-practice of NPs, were shown to increase healthcare utilization for rural and vulnerable patients, among others [18]. In its decentralized regulatory system, NPs showed an increased odds to work in primary care than specialty practices in those states with no restrictions to scopes-of-practice laws [19]. There are reportedly still regulatory barriers for NPs restricting their potential in reducing health inequalities in the U.S. [20].

The article by Nissanholtz-Gannot et al. [1] does not go into detail on the role of policy and regulation in the uptake of new roles among community nurses in Israel. In other countries, scopes-of-practice laws or bylaws that list individual tasks at a granular level have been shown to be inflexible and time-consuming to change [4, 21]. Countries with substantial changes to the skills and roles of their nursing professions should regularly revisit the specific scopes-of-practice, ideally by independent experts, as was done in the Netherlands [2].

### Financing, payment and labor markets

In Israel, nurses reported that financing and payment policies negatively impacted on the uptake of their roles. In particular, low compensation levels, the lack of resources and insufficient job positions were identified as critical barriers to nurses working in expanded roles [1].

These findings are mirrored by other countries worldwide. Educating nurses in expanded roles is not sufficient if health labor markets are inflexible and do not adjust to take advantage of these skill-mix changes [22, 23]. In countries in which few new job positions were created or where insufficient funding was available, the uptake of new nursing roles has shown to be limited [2]. No compensation or low payment levels not taking into account nurses’ expanded roles, have been shown to negatively affect the uptake of expanded nursing roles. Conversely, in Estonia and Lithuania for instance, financial incentives were used to accelerate the implementation of expanded nursing roles in primary care practices [2]. Implementing skill-mix change should not only be aligned with reforms to the educational sector, but also undertaken in line with a rigorous, in-depth analysis of the labor market, payment policies and workforce planning.

To date, there is very limited research on the role of labor markets on the uptake of nurses’ expanded roles with a particular focus on health promotion and prevention activities. The design and use of payment policies and financial incentives should be evaluated as to intended and unintended consequences in stepping up individual health promotion activities, particularly for vulnerable population groups.

## Conclusions

In response to changing patient needs, health workforce skill-mismatches and a shift from hospital to primary care, nurses are taking on a larger role in the community in Israel and worldwide. Changes to nurses’ roles are often targeted at providing more comprehensive services to patients with chronic conditions and for vulnerable, high risk groups. Several factors play a role in the implementation of these nurse skill-mix changes if the policy aim is to achieve the full effectiveness of these new roles: removing regulatory barriers to practice, revisiting training and education, adjusting financing and payment policies in line with the required skills as well as creating sufficient job positions.

## Abbreviations

AIDS: Acquired Immune Deficiency Syndrome
APN: Advanced Practice Nurse
HIV: Human Immunodeficiency Virus
NHI: National Health Insurance
NP: Nurse Practitioner
U.S.: United States of America
